# Distinguishing Patients With Distant Metastatic Differentiated Thyroid Cancer Who Biochemically Benefit From Next Radioiodine Treatment

**DOI:** 10.3389/fendo.2020.587315

**Published:** 2020-11-16

**Authors:** Ri Sa, Lin Cheng, Yuchen Jin, Hao Fu, Yan Shen, Libo Chen

**Affiliations:** ^1^ Department of Nuclear Medicine, Shanghai Jiao Tong University Affiliated Sixth People’s Hospital, Shanghai, China; ^2^ Department of Nuclear Medicine, The First Hospital of Jilin University, Changchun, China; ^3^ Department of Radiology, Shanghai Chest Hospital, Shanghai Jiao Tong University, Shanghai, China

**Keywords:** thyroid cancer, radioiodine, target/background ratio, thyroglobulin, biochemical response

## Abstract

**Background:**

Repeated radioiodine (^131^I) treatment (RT) are commonly performed in patients with ^131^I-avid distant metastatic differentiated thyroid cancer (DM-DTC), but more precise indications remain indeterminate. This prospective study was conducted to explore predictors for biochemical response (BR) to next RT.

**Methods:**

Totally thyroidectomized patients with ^131^I-avid DM-DTC demonstrated by initial post-therapeutic whole body scan (Rx-WBS) were consecutively recruited. Repeated RTs were performed at a fixed dose and a fixed interval, which was terminated once a decline in thyroid stimulating hormone-suppressed thyroglobulin (Tg_on_) could not be achieved or Rx-WBS was negative. BR was evaluated by change rate of Tg_on_ level (ΔTg_on_%).

**Results:**

After exclusion of 27 ineligible courses, a total of 166 neighboring course pairs from 77 patients were established and utilized. Univariate and multivariate analyses showed that the maximum target/background ratio (T/B_max_) on the whole body scan and ΔTg_on_% derived from the former RT were independently associated to the latter one. In predicting biochemical remission, the positive predictive value (PPV) and negative predictive value (NPV) of T/B_max_ at the cut-off value of 8.1 were 79.1% and 84.0%, respectively; whereas the PPV and NPV of ΔTg_on_% at the cut-off value of 25.3% were 70.8% and 77.1%, respectively. Notably, the PPV of combined T/B_max_ ≥ 8.1 and ΔTg_on_% ≥ 25.3% increased to 87.7%; while the NPV of T/B_max_ ≥ 8.1 or ΔTg_on_% ≥ 25.3% reached as high as 97.7%.

**Conclusions:**

This study revealed that combined use of the latest RT-derived T/B_max_ and ΔTg_on_% may efficiently identify biochemical responders/non-responders to next RT, warranting management optimization of patients with ^131^I-avid DM-DTC.

## Introduction

Papillary thyroid cancer and follicular thyroid cancer are collectively termed as differentiated thyroid cancer (DTC), which accounts for approximately 90% of thyroid cancer, and are generally indolent with relatively favorable prognosis (1, 2). Distant metastatic differentiated thyroid cancer (DM-DTC) occupying 4%–15% of all DTC cases at initial presentation or during subsequent follow-up, however, represents the main cause of disease-specific mortality (3). Traditionally targeted therapy using radioiodine (^131^I) with multiple courses has become a routine therapeutic strategy for ^131^I-avid DM-DTC for decades, with regard to ^131^I uptake demonstrated by post-therapeutic whole body scan (Rx-WBS) (4).

As is well known that the 2015 American Thyroid Association (ATA) management guidelines provide only general indication for repeated ^131^I treatment (RT) by suggesting that ^131^I-avid metastatic lesions may be treated with ^131^I and that RT may be repeated when an objective benefit is demonstrated (5). Due to difficulties in a detailed definition of so-called “objective benefit”, the guidelines failed to describe more precise indications for repeated RT of ^131^I-avid DM-DTC. Many centers continue to simply repeat RT as long as there is visible ^131^I-avid lesions on Rx-WBS and/or elevated thyroglobulin (Tg) (6), yielding a potential overuse of ^131^I in patients with radioiodine-refractory disease (7) and increased risk of side effects, such as salivary gland dysfunction (8), pulmonary fibrosis (9), bone marrow suppression (10), and secondary cancers (11). More importantly, an objective benefit from a latest course of RT does not necessarily mean that patient continues to benefit from next course, as varying responses to repeated RT have been noted in previous studies (12, 13).

Additionally, since patient-based retrospective qualitative studies merely demonstrate multiple factors possibly related to final outcome of accumulated RTs, they hardly provide more reliable evidences for or against next RT (14–16). We, therefore, conducted this prospective course-based study to identify predictors for biochemical response (BR) to next RT from clinicopathological features in patients with ^131^I-avid DM-DTC followed by efficacy assessment, aiming at optimizing indications for repeated RT and reducing potential abuse of ^131^I.

## Materials and Methods

### Study Conduct

All the patients provided written informed consent prior to the initiation of the study. The protocol was approved by the Ethics Committee of Shanghai Jiao Tong University Affiliated Sixth People’s Hospital (Shanghai, P.R. China). The authors vouched for the completeness and accuracy of the data and analyses.

### Study Populations

Totally thyroidectomized patients with ^131^I-avid DM-DTC demonstrated by initial Rx-WBS in a single institution were consecutively recruited from May 1^st^, 2014. Following low-iodine diet and levothyroxine withdrawal for 4 weeks as in preparation for initial RT, repeated RT was performed at a fixed ^131^I dose of 7.4 GBq with an interval of nearly 6 months between neighboring therapeutic ^131^I administrations (5, 17). RT was terminated once a decline in thyroid stimulating hormone (TSH)-suppressed Tg (Tg_on_) could not be achieved or Rx-WBS was negative. Nearly 1 month after RT, patients were visited and TSH, free triiodothyronine, and free thyroxine were examined to guide the adjustment of levothyroxine dosage.

### Establishment of Eligible RT Course Pair

A course pair was constituted by neighboring two RTs (the former course and the latter course). For example, if a patient underwent three courses of RT, the second course acted as not only the latter course of the first course pair but also the former course of the second course pair, and so on. ^131^I-avid DM-DTC and baseline Tg_on_ level just before former RT ≥ 10.0 ng/ml were the enrollment criteria of course pair. Course pairs possibly interfered by cancers beyond DTC, other therapeutics beyond RT, suppressed TSH level > 0.1 mIU/L (5), and anti-Tg antibody (TgAb) > 60.0 IU/ml were excluded. Eligible course pairs were then utilized in the univariate and multivariate analyses to identify factors associated with biochemical response (BR) to next RT.

### Serologic Examinations

TSH, Tg, and TgAb levels were measured before, one and 4 months after RT by an electrochemiluminescent immunoassay on a Cobas analyzer (Roche Diagnostics Gmbh, Roche Ltd., Basel, Switzerland). The lower and upper detection limits of the TSH assay were 0.005 and 100 mIU/L, respectively, and TSH levels lower or higher than those were counted as 0.005 and 100 mIU/L, respectively. Similarly, Tg levels higher than 50,000 ng/ml were counted as 50,000 ng/ml, while TgAb levels lower than 10 IU/ml were counted as 10 IU/ml.

### Post-Therapeutic Whole Body Scan

Rx-WBS was obtained 3 days after the oral administration of 7.4 GBq of ^131^I in liquid by a single photon emission computed tomography/computed tomography (SPECT/CT, GE Discovery NM/CT 670) equipped with detectors composed of NaI(Tl) crystal with 3/8 inch thickness and parallel-hole high-energy collimators. Anterior and posterior acquisitions were simultaneously performed at a speed of 10 cm/min. The body-contouring system was used to minimize the distance between the patient and the collimator. A low dose CT (voltage of 120keV) was added immediately to yield SPECT/CT images if Rx-WBS had shown inconclusive findings to achieve definitive diagnoses as previously described by our team (18). Semi-quantitative analysis of ^131^I uptake of metastatic foci in planar imaging was conducted using a region-of-interest (ROI) technique (19). Briefly, regions were drawn separately around each distant metastatic lesion and normal frontal cranial bone on anterior and posterior images, and the maximum count of each ROI was obtained and utilized to yield the maximum target/background ratio (T/B_max_) in each Rx-WBS, representing the only one most active metastatic lesion.

### Biochemical Response Assessment

BR was evaluated by the comparison between post-therapeutic Tg_on_ approximately 4 months post RT and pre-therapeutic Tg_on_ just before levothyroxine withdrawal. ΔTg_on_%, change rate of Tg_on_ level, was defined as follows: [(pre-therapeutic Tg_on_ level–post-therapeutic Tg_on_ level)/pre-therapeutic Tg_on_ level] × 100%.

In determining the categorization of BR to the latter course of RT, the following standards were used:

ΔTg_on_% ≥ 25.0% indicated effective RT (biochemical remission), while ΔTg_on_% < 25.0% meant non-effective RT, including biochemical stabilization (-25.0% ≤ ΔTg_on_% < 25.0%) and biochemical progression (ΔTg_on_% < -25.0%) (4, 20).

### Statistical Analysis

Continuous variables with normal distributions are presented as means ± standard deviations, whereas continuous variables with non-normal distribution are presented as medians with ranges. Categorical variables are reported as numbers with percentages. Unpaired Student’s *t*-tests, Mann-Whitney U-tests, Fisher’s tests, or chi-square tests were used for univariate analyses as needed, and significant variables were included in the subsequent multivariate logistic regression analysis. The optimum cut-off values for independent predictors were calculated using receiver operating characteristic (ROC) curves. Statistical analyses were performed using SPSS software (v. 24.0). All *p* values were two-sided; *p* values < 0.05 were considered statistically significant.

## Results

### Clinical Characteristics

From May 1^st^, 2014 through November 31^st^, 2018, a total of 193 course pairs were consecutively enrolled. After exclusion of 8 course pairs interfered by cancers beyond DTC, 6 course pairs interfered by other therapeutics including operation, radiotherapy, targeted therapy, and 13 course pair with inappropriate suppressed TSH level or elevated TgAb, a total of 166 eligible course pairs from 77 patients (age, 42.4 ± 11.5 years) were finally established and utilized.

Regarding the continuous variables of the 166 former courses of interest, the median baseline Tg_on_ and TSH-stimulated Tg (Tg_off_) levels just before RT were 199.0 ng/ml (range, 11.2–29,980.0 ng/ml) with a median TSH of 0.04 mIU/L (range, 0.005–0.07 mIU/L) and 1116.0 ng/ml (range, 35.4–50,000.0 ng/ml) with a median TSH of 100.0 mIU/L (range, 33.8–100.0 mIU/L), respectively. At 4 months post RT, the median Tg_on_ declined to 134.0 ng/ml (range, 10.3–22,498.0 ng/ml) with a median TSH of 0.06 mIU/L (range, 0.005–0.09 mIU/L), yielding a median ΔTg_on_% of 31.7% (range, -367.3%–96.8%). The median T/B_max_ was 11.0 (range, 1.0–317.5). Besides, the categorical clinicopathological characteristics of eligible former courses of RT from 77 patients are shown in **Table 1**.

**Table 1:** Categorical clinicopathological characteristics of eligible former ^131^I therapeutic courses (N = 166) from 77 patients.

Characteristics	Patient-n (%)	Course-n (%)
Gender		
Female	47 (61.0)	95 (57.2)
Male	30 (39.0)	71 (42.8)
Histologic subtype of DTC		
Papillary	60 (77.9)	123 (74.1)
Follicular or mixed	17 (22.1)	43 (25.9)
T stage		
T1-2	20 (26.0)	21 (12.7)
T3-4	57 (74.0)	145 (87.3)
N stage		
N0	2 (2.6)	4 (2.4)
N1	75 (97.4)	162 (97.6)
ETE		
Yes	54 (70.1)	136 (81.9)
No	23 (29.9)	30 (18.1)
Thyroid remnant visualized on Rx-WBS		
Yes	37 (48.1)	52 (31.3)
No	40 (51.9)	114 (68.7)
Radioactive iodine uptake 4 weeks after levothyroxine withdrawal		
≤2%	38 (49.3)	92 (55.4)
>2%–5%	29 (37.7)	64 (38.6)
>5%–15%	8 (10.4)	8 (4.8)
>15%	2 (2.6)	2 (1.2)
Involved organ		
Single	66 (85.7)	94 (56.7)
Multiple	11 (14.3)	72 (43.3)
Sites of distant metastasis		
Only lung	50 (64.9)	107 (64.5)
Only bone	12 (15.6)	27 (16.3)
Combined or others	15 (19.5)	32 (19.2)
Metastatic lesions on Rx-WBS		
Single	16 (20.8)	21 (12.6)
Multiple	61 (79.2)	145 (87.4)
^131^I-nonavid metastases concurrent with ^131^I-avid metastases on Rx-WBS		
Yes	9 (11.7)	19 (11.5)
No	68 (88.3)	147 (88.6)
Accumulated ^131^I activity		
<22.2 GBq	46 (59.7)	114 (68.7)
≥22.2 GBq	31 (40.3)	52 (31.3)

DTC, differentiated thyroid cancer; T, tumor; N, lymph node; ETE, extrathyroidal extension; Rx-WBS, post-therapeutic whole body scan.

Out of 166 paired latter courses, effective RT (biochemical remission) was obtained in 84 (50.6%) courses; while 49.4% courses yielded non-effective RT, including biochemical stabilization and biochemical progression in 30.2% and 19.2% of all courses, respectively.

### Identification of Predictors for BR to Next RT

Upon analyzing the association of potential clinicopathologic features with BR to next RT, a total of 16 former course-derived factors were involved in the univariate analysis. We found that thyroid remnant visualized on Rx-WBS, ΔTg_on_%, T/B_max_, and T stage were associated with BR (*p* < 0.05). In the multivariable logistic regression analysis, however, only T/B_max_ and ΔTg_on_% were remained as independent predictors for biochemical remission with Odds ratios of 3.809 and 1.044, respectively (**Table 2**).

**Table 2:** Univariate analysis of factors potentially associated with biochemical response to next radioiodine treatment in 166 former courses.

Variable	Effective RT n = 84	Non-effective RT n = 82	Univariate analysis		Multivariate analysis	
			*P*	OR (95% CI)	*P*	OR (95% CI)
Age	42.9 ± 16.1	41.9 ± 16.9	0.857	1.002 (0.997–1.028)	–	–
Gender-n (%)			0.928	1.039 (0.449–2.408)	–	–
Female	49 (58.3)	46 (56.1)				
Male	35 (41.7)	36 (43.9)				
Histologic subtype of DTC-n (%)			0.465	0.684 (0.247–1.896)	–	–
Papillary	61 (72.6)	62 (75.6)				
Follicular or mixed	23 (27.4)	20 (23.2)				
T stage-n (%)			0.017	5.513 (1.357–22.396)	0.103	2.597 (0.825–8.171)
T1-2	15 (17.9)	6 (7.3)				
T3-4	69 (82.1)	76 (92.7)				
N stage-n (%)			0.083	21.566 (0.692–692.0)	–	–
N0	3 (0.2)	1 (0.2)				
N1	83 (98.8)	81 (98.8)				
ETE-n (%)			0.577	0.749 (0.272–2.067)	–	–
Yes	71 (84.5)	65 (79.3)				
No	13 (15.5)	17 (20.7)				
Tg_on_ before RT-median (range, ng/ml)	261.6 (12.8–29974.2)	178.5 (11.4–20473.0)	0.188	1.000 (1.000–1.000)	–	–
Tg_off_ before RT-median (range, ng/ml)	997.0 (35.4–50000.0)	1101.0 (54.9–25000.0)	0.832	1.000 (1.000–1.000)	–	–
Tg_on_ at 4 months after RT-median (range, ng/ml)	111.7 (10.5–20473.0)	141.3 (10.3–22498.0)	0.098	1.000 (0.999–1.000)	–	–
ΔTg_on_%-median (range)	40.3% (-367.3–97.4)	7.9% (-819.6–93.7)	0.042	2.546 (1.033–6.279)	0.001	3.809 (1.724–8.814)
Thyroid remnant visualized on Rx-WBS-n (%)			0.042	0.410 (0.174–0.968)	0.056	1.751 (0.216–1.020)
Yes	33 (39.3)	19 (23.2)				
No	51 (60.7)	63 (76.8)				
Involved organ-n (%)			0.451	0.695 (0.270–1.789)	–	–
Single	47 (56.0)	47 (57.3)				
Multiple	37 (44.1)	35 (42.7)				
Metastatic lesions on Rx-WBS-n (%)			0.692	0.783 (0.234–2.619)	–	–
Single	10 (11.9)	11 (13.4)				
Multiple	74 (88.1)	71 (86.6)				
^131^I-nonavid metastases concurrent with ^131^I-avid metastases on Rx-WBS-n (%)			0.177	2.442 (0.669–8.914)	–	–
Yes	6 (7.1)	13 (15.9)				
No	78 (92.9)	69 (84.1)				
T/B_max_-median (range)	15.1 (1.4–317.8)	6.00 (1.0–72.5)	0.006	1.044 (1.012–1.077)	0.003	1.044 (1.015–1.073)
Accumulated ^131^I activity-median (range, GBq)	12.9 (7.4–44.4)	18.5 (7.4–59.2)	0.255	0.999 (0.998–1.001)	–	–

DTC, differentiated thyroid cancer; OR, odds ratio; T, tumor; N, lymph node; ETE, extrathyroidal extension; Tg_on_, thyroid stimulating hormone-suppressed thyroglobulin; Tg_off_, thyroid stimulating hormone-stimulated thyroglobulin; ΔTg_on_%, change rate of Tg_on_ level; RT, radioiodine treatment; Rx-WBS, post-therapeutic whole body scan; T/B_max_, maximum target/background ratio.

### Efficacy of T/B_max_ and/or ΔTg_on_%

Cut-off values of T/B_max_ at 8.1 and ΔTg_on_% at 25.3% were obtained by ROC analyses to optimally differentiate effective RT from non-effective RT. The predictive efficacy of a T/B_max_ of 8.1 and/or ΔTg_on_% of 25.3% were further calculated and are listed in **Table 3**. The specificity of T/B_max_ ≥ 8.1 and ΔTg_on_% ≥ 25.3% was 90.2%, and the sensitivity of T/B_max_ ≥ 8.1 or ΔTg_on_% ≥ 25.3% was 98.8%. Moreover, the positive predictive value (PPV) and negative predictive value (NPV) of T/B_max_ ≥ 8.1 were higher than those of ΔTg_on_% ≥ 25.3% (79.1% vs. 70.8%; 84.0% vs. 77.1%). Notably, the highest PPV of 87.7% was achieved by a criterion of combined T/B_max_ ≥ 8.1 and ΔTg_on_% ≥ 25.3%, whereas a criterion of T/B_max_ ≥ 8.1 or ΔTg_on_% ≥ 25.3% yielded the highest NPV of 97.7%.

**Table 3:** Efficacy of T/B_max_ of 8.1 and/or ΔTg_on_% of 25.3% in predicting biochemical response to next radioiodine treatment.

Criterion	Sen (%)	Spe (%)	PPV (%)	NPV (%)
T/B_max_ ≥ 8.1	85.7	76.8	79.1	84.0
ΔTg_on_% ≥ 25.3%	80.9	65.9	70.8	77.1
T/B_max_ ≥ 8.1 and ΔTg_on_% ≥ 25.3%	67.9	90.2	87.7	73.3
T/B_max_ ≥ 8.1 or ΔTg_on_% ≥ 25.3%	98.8	52.4	68.0	97.7

T/B_max_, maximum target/background ratio; Tg_on_, thyroid stimulating hormone-suppressed thyroglobulin; ΔTg_on_%, change rate of Tg_on_ level; Sen, sensitivity; Spe, specificity; PPV, positive predictive value; NPV, negative predictive value.

### Application of T/B_max_ of 8.1 Combined With ΔTg_on_% of 25.3%

**Figure 1:**
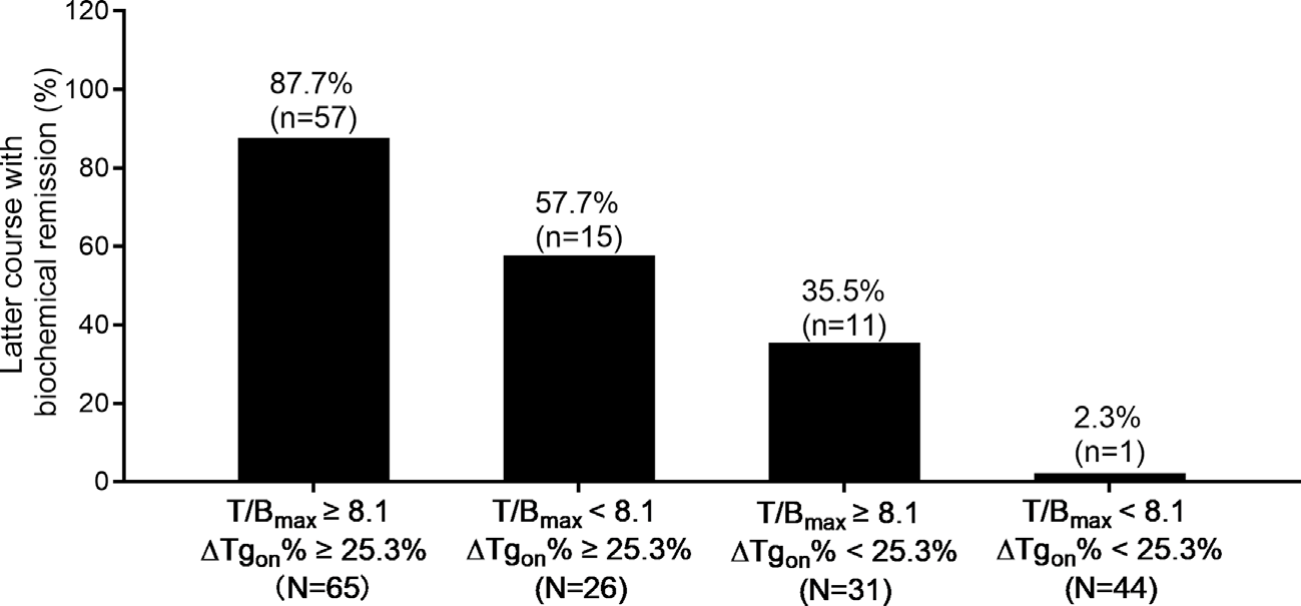
Distribution of biochemical remission in 166 paired latter courses of RT with regard to the results of T/B_max_ and ΔTg_on_% obtained from the former courses. RT, radioiodine treatment; T/B_max_, maximum target-to-background ratio; Tg_on_, thyroid stimulating hormone-suppressed thyroglobulin; ΔTg_on_%, change rate of Tg_on_ level.

**Figure 2:**
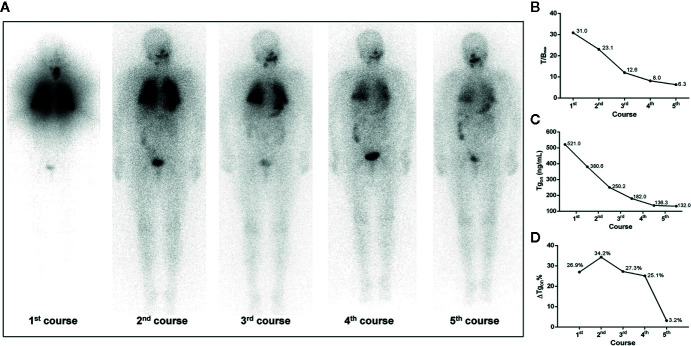
A 68-year-old female differentiated thyroid cancer patient with ^131^I-avid pulmonary metastases responding to RT. The T/B_max_ and ΔTg_on_% resulting from the first, second, and third course of RT were more than 8.1 (31.0, 23.1, and 12.6) and 25.3% (26.9%, 34.2%, and 27.3%), respectively, indicating biochemical remission was achievable in the paired latter courses, which were confirmed by the significant decrease in Tg_on_ resulting from the second, third, and fourth course of RT, respectively. After the fourth administration of ^131^I, nevertheless, T/B_max_ decreased to 8.0 and ΔTg_on_% declined to 25.1%, indicating a non-effective RT was achievable in the paired latter courses, which was confirmed by the subtle decrease in Tg_on_ (3.2%) resulting from the fifth course of RT. **(A)** Whole body scans (anterior) at 3 days after the oral administration of 7.4 GBq of ^131^I; **(B)** T/B_max_; **(C)** Tg_on_; **(D)** ΔTg_on_%. RT, radioiodine treatment; T/B_max_, maximum target-to-background ratio; Tg_on_, thyroid stimulating hormone-suppressed thyroglobulin; ΔTg_on_%, change rate of Tg_on_ level.

**Figure 3:**
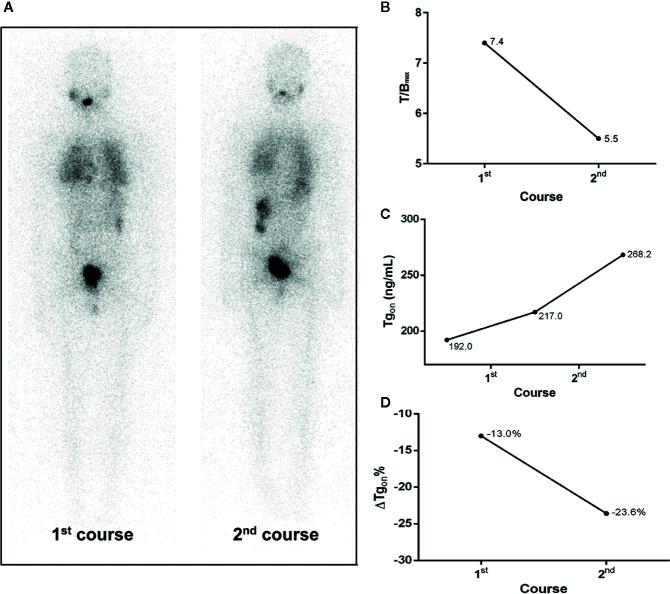
A 20-year-old female differentiated thyroid cancer patient with ^131^I-avid pulmonary metastases not responding to RT. The T/B_max_ and ΔTg_on_% resulting from the initial course of RT were less than 8.1 and 25.3%, respectively, indicating a non-effective RT was achievable in the paired latter courses, which was confirmed by the increase in Tg_on_ (23.6%) at 4 months after the second course of RT. **(A)** Whole body scans (anterior) at 3 days after the oral administration of 7.4 GBq of ^131^I; **(B)** T/B_max_; **(C)** Tg_on_; **(D)** ΔTg_on_%. RT, radioiodine treatment; T/B_max_, maximum target-to-background ratio; Tg_on_, thyroid stimulating hormone-suppressed thyroglobulin; ΔTg_on_%, change rate of Tg_on_ level.

**Figure 4:**
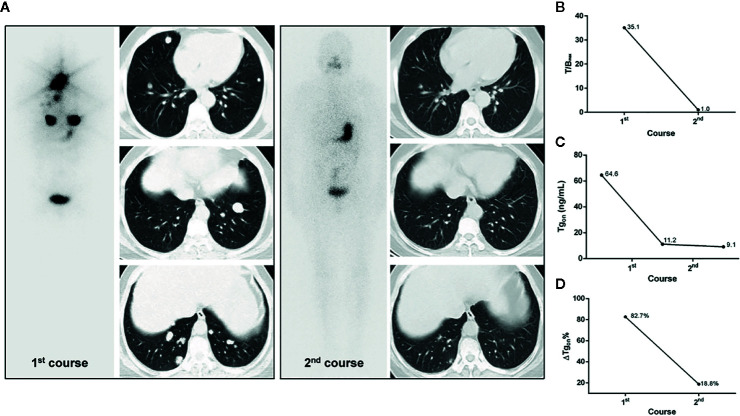
A 53-year-old female differentiated thyroid cancer patient with robust ^131^I-avid pulmonary metastases achieving excellent benefit from the initial RT. The T/B_max_ and ΔTg_on_% resulting from the initial course of RT were 35.1 and 82.7%, respectively. In the second course of RT, unexpectedly, post therapeutic whole body scans were negative and the size of lesions were dramatically decreased on chest computed tomography, along with a Tg_on_ of 9.1 ng/ml, indicating an excellent benefit from the initial course of RT, which was demonstrated by a continuous decrease in Tg_on_ to 0.9 ng/ml during the 3-year follow-up. **(A)** Whole body scans (anterior) at 3 days after the oral administration of 7.4 GBq of ^131^I; **(B)** T/B_max_; **(C)** Tg_on_; **(D)** ΔTg_on_%. RT, radioiodine treatment; T/B_max_, maximum target-to-background ratio; Tg_on_, thyroid stimulating hormone-suppressed thyroglobulin; ΔTg_on_%, change rate of Tg_on_ level.

According to the results of T/B_max_ and ΔTg_on_% obtained from the 166 former courses and the categorical criteria (T/B_max_ ≥ 8.1 and ΔTg_on_% ≥ 25.3%, T/B_max_ ≥ 8.1 and ΔTg_on_% < 25.3%, T/B_max_ < 8.1 and ΔTg_on_% ≥ 25.3%, and T/B_max_ < 8.1 and ΔTg_on_% < 25.3%), the BR distribution of the 166 paired latter courses are shown in **Figure 1**. Upon the 166 latter courses, consistent (T/B_max_ ≥ 8.1 and ΔTg_on_% ≥ 25.3%; T/B_max_ < 8.1 and ΔTg_on_% < 25.3%) and inconsistent (T/B_max_ ≥ 8.1 and ΔTg_on_% < 25.3%; T/B_max_ < 8.1 and ΔTg_on_% ≥ 25.3%) conditions were found in 109 (65.7%) and 57 (35.3%) courses, respectively, with biochemical remission rates of 53.2% (58/109) and 45.6% (28/57), respectively. Representative cases with effective RT or non-effective RT are illustrated in **Figures 2** and **3**, respectively. Additionally, four patients with T/B_max_ ≥ 8.1 and ΔTg_on_% ≥ 25.3% who obtained excellent benefits from the former RT exhibited subtle Tg_on_ levels before and negative Rx-WBS after the latter course of RT (**Figure 4**).

**Figure 5:**
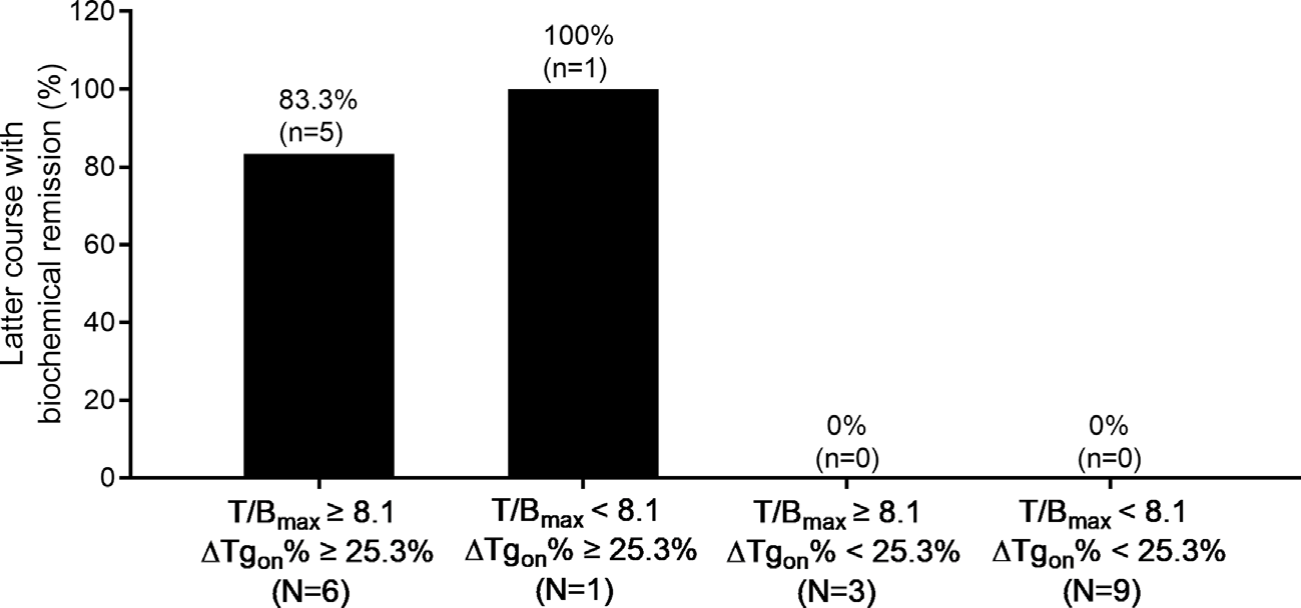
Distribution of biochemical remission in 19 paired latter courses of RT in patients with ^131^I-nonavid metastases concomitant with ^131^I-avid metastases with regard to the results of T/B_max_ and ΔTg_on_% obtained from the former courses. RT, radioiodine treatment; T/B_max_, maximum target-to-background ratio; Tg_on_, thyroid stimulating hormone-suppressed thyroglobulin; ΔTg_on_%, change rate of Tg_on_ level.

Additionally, ^131^I-nonavid metastasis concurrent with ^131^I-avid metastasis was found in 11 patients with a total of 19 former courses. In this entity, 83.3% (5/6) of the latter courses of RT yielded biochemical remission if T/B_max_ ≥ 8.1 and ΔTg_on_% ≥ 25.3% were met in the former course, while no biochemical remission (0/9) was obtained from the latter RT course if the former course of RT created T/B_max_ < 8.1 and ΔTg_on_% < 25.3%. The distribution of biochemical remission in all the paired latter courses is shown in **Figure 5**.

**Figure 6:**
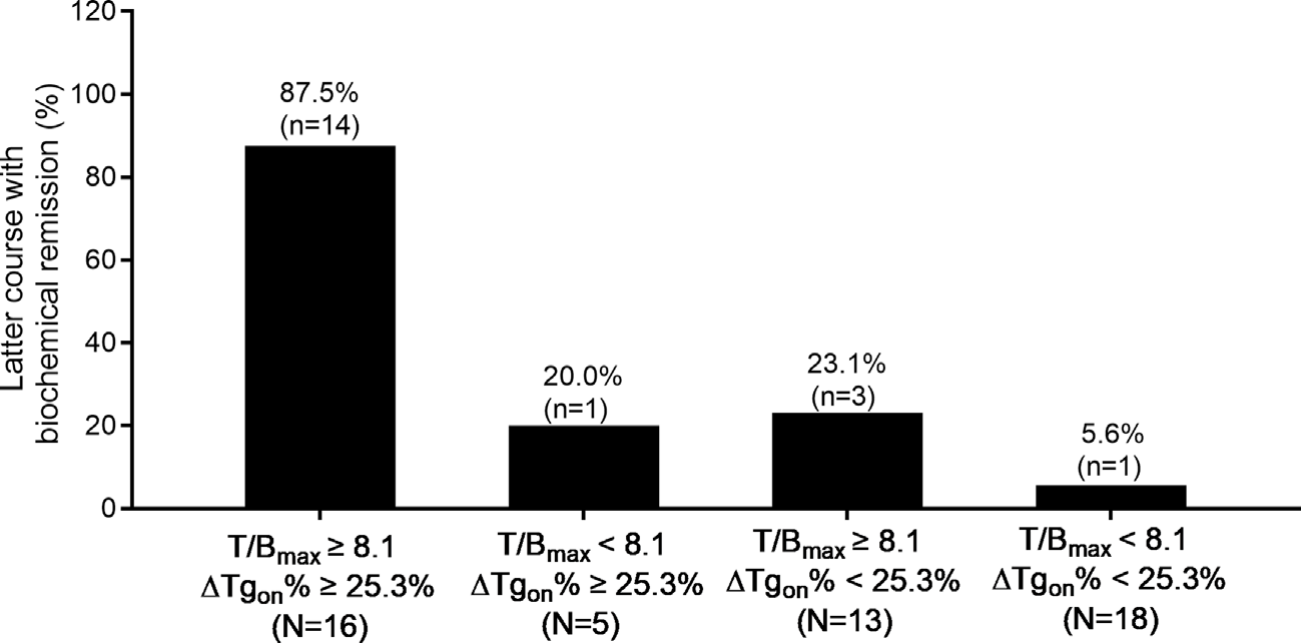
Distribution of biochemical remission in 52 paired latter courses of RT in patients with accumulated ^131^I activity ≥ 22.2 GBq with regard to the results of T/B_max_ and ΔTg_on_% obtained from the former courses. RT, radioiodine treatment; T/B_max_, maximum target-to-background ratio; Tg_on_, thyroid stimulating hormone-suppressed thyroglobulin; ΔTg_on_%, change rate of Tg_on_ level.

Moreover, accumulated ^131^I activity ≥ 22.2 GBq was administered in 20 patients with a total of 52 former courses. In this entity, 87.5% (14/16) of the latter courses of therapy brought biochemical remission if T/B_max_ ≥ 8.1 and ΔTg_on_% ≥ 25.3% were met in the former course, while only 5.6% (1/18) of the latter courses of RT resulted in biochemical remission if the former course of RT had yielded in T/B_max_ < 8.1 and ΔTg_on_% < 25.3%. The distribution of biochemical remission in the paired latter courses is shown in **Figure 6**.

## Discussion

In the era of precision medicine, a justification of RT is critical for the management of DTC, especially in patients with ^131^I-avid DM-DTC who possibly need multiple RT courses. Optimization of indications is a key step to enhance therapeutic efficacy and avoid inadequate or invalid ^131^I administrations, and appropriate predictors for responders/non-responders are anticipated to distinguish patients who would receive next RT from those who should turn to other management timely. To our knowledge, this is the first prospective quantitative study to predict BR to next RT by combined use of T/B_max_ and ΔTg_on_% derived from the latest RT, providing a practical evidence for or against next RT in a course-based manner, warranting the justification of repeating RT or not.

Since that Rx-WBS reveals functioning metastases secondary to DTC, positive findings have been used to guide subsequent RT (21, 22). However, ^131^I uptake reflected by the latest Rx-WBS does not necessarily mean that the patient will respond to next RT due to a neglect of absorbed radiation dose to metastatic lesions, and the PPV of Rx-WBS in predicting final beneficial BR has been reported to be only 60.0% in patients with DM-DTC (23), which is in line with our present study demonstrating that a high rate as 49.4% (82/166) of latter RTs failed to achieve biochemical remission (**Figure 1**). Of note, T/B_max_ derived from the Rx-WBS of the former RT was found to be an independent predictor with PPV and NPV of 79.1% and 84.0% at the cut-off value 8.1, respectively. It means that a higher T/B_max_ obtained from the former Rx-WBS is associated with biochemical remission.

Tg, a reliable DTC-specific marker, positively correlates with tumor burden in totally thyroidectomized patients. Tg_on_ > 10.0 ng/ml may be sufficient to ensure the existence of DTC in patients who have undergone total thyroidectomy (24, 25), which became one of the two enrollment criteria in our analysis. Unexpectedly, neither baseline Tg_off_ nor baseline Tg_on_ of the former RT were associated with BR to the latter RT, whereas ΔTg_on_% turned out to be an independent predictor. It seems that a higher ΔTg_on_% derived from the former course correlates with biochemical remission. Unfortunately, predictive value of the ΔTg_on_% was relatively limited, as the PPV and NPV of ΔTg_on_% at 25.3% in our study were merely 70.8% and 77.1%, respectively (**Table 3**). This may be due to that synthesis of Tg does not accurately reflect the iodine consumption in DM-DTC lesions, which lays critical foundation for biochemical remission (26).

As mentioned above, the value of T/B_max_ or ΔTg_on_% alone in predicting BR to the next course of RT was not sufficiently high. Inspiringly, the combined use of semi-quantitative T/B_max_ and quantitative ΔTg_on_% displayed a robustly enhanced PPV and NPV compared with that of either T/B_max_ or ΔTg_on_% alone. In the present study, T/B_max_ ≥ 8.1 and ΔTg_on_% ≥ 25.3% possessed the best PPV, while T/B_max_ ≥ 8.1 or ΔTg_on_% ≥ 25.3% yielded the optimal NPV (**Table 3**). These findings suggest that biochemical response to next RT can be confidently expected in patients whose latest RT has yielded a T/B_max_ ≥ 8.1 and a ΔTg_on_% ≥ 25.3%, whereas patients whose latest RT has yielded a T/B_max_ < 8.1 and a ΔTg_on_% < 25.3% would hardly benefit from next RT. Such stratification based on T/B_max_ combined with ΔTg_on_% derived from the latest RT may be useful in justifying the acceptance or refusal of the next course. By the way, difficulties in predicting BR were encountered in approximately one third of former courses, implying that other predictors are still needed in decision-making of such inconsistent situations (T/B_max_ ≥ 8.1 and ΔTg_on_% < 25.3%, or T/B_max_ < 8.1 and ΔTg_on_% ≥ 25.3%).

Notably, excellent benefits were achieved in four former RT courses in the group of T/B_max_ ≥ 8.1 and ΔTg_on_% ≥ 25.3%. Tg_on_ levels subtly higher than 10.0 ng/ml and negative Rx-WBS findings were found in these four patients before and after RT, respectively. Such excellent benefits from former courses may compromise the PPV of T/B_max_ ≥ 8.1 and ΔTg_on_% ≥ 25.3%, to a certain degree. This might be due to that a greater than 25% decrease in Tg_on_ was hardly achievable by the latter RT, since the tumor burden after the initial RT was relieved substantially or even disappeared (**Figure 4**).

Moreover, in patients with ^131^I concentrations in some foci but not in others, 83.33% (5/6) of the courses of RT brought biochemical remission if the criterion of T/B_max_ ≥ 8.1 and ΔTg_on_% ≥ 25.3% was met (**Figure 5**), indicating that such patients should not be hastily labelled as radioiodine refractoriness and repeated RT should not be arbitrarily terminated (7). Conversely, a T/B_max_ < 8.1 and ΔTg_on_% < 25.3% in this setting implies that further RT should be avoided or a ^131^I dose of 7.4 GBq may be insufficient. Similar findings were obtained in patients with accumulated ^131^I activity over 22.2 GBq (**Figure 6**). Therefore, the combined use of both predictors with optimal cut-off values establishes an indispensable tool for decision-making in the above both entities.

It is well known that the role of RT may be influenced by adopted efficacy assessment criteria. For instance, the routinely used Response Evaluation Criteria in Solid Tumors are usually impractical in the assessment of responses to RT in DM-DTC patients, as non-measurable lesions are commonly found (27–29). Moreover, morphological changes of lesions are not easily perceived by anatomical imaging during a relatively short interval, since ^131^I-avid DM-DTC is well differentiated and indolent in nature, and repeated RT is recommended to be administered every 6–12 months. Fortunately, Tg level is closely associated with prognosis of patients with DM-DTC irrespective of age, initial staging and risk-stratification (30) and the ΔTg_on_% has been confirmed to reflect the response to RT and the degree of tumor destruction (31). In previous studies, it was well accepted that the ΔTg_on_% thresholds of 20.0% and 25.0% were adopted for differentiating effective RT from non-effective RT (4, 32). To utmostly avoid the invalid RT, the threshold ΔTg_on_% of 25.0% was adopted in our study to screen clinicopathological features associated with biochemical remission. Moreover, it is noteworthy that, along with biochemical progression, we categorized biochemical stabilization into non-effective RT, owing to that DTC is an indolent tumor with a long duration of stable Tg level under TSH suppression. Thus, prospective randomized controlled studies are required to elucidate whether stable BR truly represents a benefit of RT.

Some limitations exist in our study. Firstly, some clinicopathological features potentially associated with BR, such as diagnostic ^131^I scan outcomes, ^18^F-FDG PET findings, and mutational statuses, were not included in the initial study design. Secondly, all patients received a dose of 7.4 GBq in each administration with an interval of nearly 6 months between neighboring courses regarding professional guidelines, and presumed impact of a former RT on the biochemical effect of the latter course could not be clearly determined and completely excluded (33, 34). Finally, T/B_max_ may vary with the time interval between the administration of ^131^I and post therapeutic scans, as well as the activity and status of administrated ^131^I. Similarly, Tg levels may fluctuate with time interval after the administration of ^131^I, as well as be interfered by the presence of TgAb and/or TSH levels. We, for the sake of extensibility of this study, encourage institutions to establish their own cut-off values of T/B_max_ and ΔTg_on_% derived from the latest RT to predict BR to the possible next RT.

## Conclusion

Our prospective study demonstrated that T/B_max_ combined with ΔTg_on_% derived from the latest course of RT may efficiently differentiate patients with ^131^I-avid DM-DTC who would biochemically benefit from next RT from those who would not, warranting management optimization of this entity.

## Data Availability Statement

The original contributions presented in the study are included in the article/supplementary materials. Further inquiries can be directed to the corresponding authors.

## Ethics Statement

The studies involving human participants were reviewed and approved by Ethics Committee of Shanghai Jiao Tong University Affiliated Sixth People’s Hospital. The patients/participants provided their written informed consent to participate in this study. Written informed consent was obtained from the individual(s) for the publication of any potentially identifiable images or data included in this article.

## Author Contributions

RS and LibC designed the study. RS, LinC, and YJ participated in patient data acquisition. RS, LinC, HF, and YS analyzed the data. RS and LibC wrote and revised the draft. All authors contributed to the article and approved the submitted version.

## Funding

This study was sponsored by the National Natural Science Foundation of China (Grant No. 81671711) and Shanghai key discipline of medical imaging (Grant No. 2017ZZ02005).

## Conflict of Interest

The authors declare that the research was conducted in the absence of any commercial or financial relationships that could be construed as a potential conflict of interest.
